# Two mechanisms of oral malodor inhibition by zinc ions

**DOI:** 10.1590/1678-7757-2017-0161

**Published:** 2018-01-16

**Authors:** Nao Suzuki, Yoshio Nakano, Takeshi Watanabe, Masahiro Yoneda, Takao Hirofuji, Takashi Hanioka

**Affiliations:** 1 Fukuoka Dental College, Department of Preventive and Public Health Dentistry, Fukuoka, Japan; 2 Nihon University, School of Dentistry, Department of Chemistry, Tokyo, Japan; 3 Fukuoka Dental College, Department of General Dentistry, Fukuoka, Japan

**Keywords:** Antimicrobial activity, Chemical binding, Hydrogen sulfide, Oral malodor, Zinc ions

## Abstract

**Objectives:**

The aim of this study was to reveal the mechanisms by which zinc ions inhibit oral malodor.

**Material and Methods:**

The direct binding of zinc ions to gaseous hydrogen sulfide (H_2_S) was assessed in comparison with other metal ions. Nine metal chlorides and six metal acetates were examined. To understand the strength of H_2_S volatilization inhibition, the minimum concentration needed to inhibit H_2_S volatilization was determined using serial dilution methods. Subsequently, the inhibitory activities of zinc ions on the growth of six oral bacterial strains related to volatile sulfur compound (VSC) production and three strains not related to VSC production were evaluated.

**Results:**

Aqueous solutions of ZnCl_2_, CdCl_2_, CuCl_2_, (CH_3_COO)_2_Zn, (CH_3_COO)_2_Cd, (CH_3_COO)_2_Cu, and CH_3_COOAg inhibited H_2_S volatilization almost entirely. The strengths of H_2_S volatilization inhibition were in the order Ag+ > Cd_2_+ > Cu_2_+ > Zn_2_+. The effect of zinc ions on the growth of oral bacteria was strain-dependent. *Fusobacterium nucleatum* ATCC 25586 was the most sensitive, as it was suppressed by medium containing 0.001% zinc ions.

**Conclusions:**

Zinc ions have an inhibitory effect on oral malodor involving the two mechanisms of direct binding with gaseous H_2_S and suppressing the growth of VSC-producing oral bacteria.

## Introduction

Oral malodor is primarily the result of microbial metabolism of amino acids from local debris in the oral cavity^19^. The primary compounds of oral malodor are volatile sulfur compounds (VSCs), such as hydrogen sulfide (H_2_S), methyl mercaptan (CH_3_SH), and dimethyl sulfide (CH_3_SCH_3_)^24^. Periodontopathic anaerobic bacteria, such as *Porphyromonas gingivalis, Treponema denticola, Prevotella intermedia, Fusobacterium nucleatum,* and *Eubacterium* can produce large amounts of H_2_S and CH_3_SH from cysteine, methionine, or serum proteins^16^ ^,^ ^17^.

Various anti-malodor agents for oral use have been introduced and have proven to be effective in reducing VSC concentration in the oral cavity. Antimicrobial agents such as chlorhexidine, triclosan, and cetylpyridinium chloride can reduce oral malodor by reducing the number of microorganisms present in the mouth^3^. Chlorine dioxide has also been shown to reduce oral malodor by chemically neutralizing VSCs^12^. Natural ingredients, such as hinokitiol, green tea powder, and *Eucalyptus* extract, also reduce oral malodor through various antibacterial mechanisms^7^ ^,^ ^11^ ^,^ ^22^.

Zinc ions are often found in commercial anti-malodor mouthwashes in combination with other active ingredients. A combination of zinc ions and chlorhexidine or cetylpyridinium chloride was reported to inhibit VSC production synergistically^29^. We considered two mechanisms of oral malodor inhibition by zinc ions. The first is that zinc ions have a strong affinity for the thiol groups present in VSCs^28^. Zinc ions exhibit immediate inhibitory effects on VSC production compared to chlorhexidine^30^, by effectively and directly reducing the activities of VSCs. The second is that zinc ions have an antibacterial effect. Zinc ions can inhibit catabolism by *F. nucleatum* and *P. intermedia* ^20^, and acid production by *Streptococcus sobrinus* and *Streptococcus salivarius* ^6^. Although these characteristics suggest that zinc ions might be effective oral anti-malodor agents, the majority of studies conducted to date have been based on the results of clinical use in combination with other agents. Therefore, the two mechanisms of action, chemical binding and an antimicrobial property, have not been individually assessed. Furthermore, previous *in vitro* studies examined the inhibitory effects of zinc ions only on the functions of selected targets, and thus the antimicrobial effects of zinc ions on microorganisms related to oral malodor remain unclear. In this study, the direct effects of zinc ions on H_2_S were assessed in comparison with other metal ions. In addition, the inhibitory effects of zinc ions on the growth of microorganisms related to VSC production and those unrelated to VSC production were evaluated.

## Materials and methods

### Direct inhibitory effects of metal ions on hydrogen sulfide

Nine metal chlorides, namely, MgCl_2_, Al_2_Cl_3_, CaCl_2_, MnCl_2_, FeCl_2_, CuCl_2_, ZnCl_2_, SrCl_2_, and CdCl_2_, and six metal acetates, namely, (CH_3_COO)_2_Ca, (CH_3_COO)_2_Fe, (CH_3_COO)_2_Cu, (CH_3_COO)_2_Zn, CH_3_COOAg, and (CH_3_COO)_2_Cd, were examined in this study. These chemical compounds, except for (CH_3_COO)_2_Cu and CH_3_COOAg, were prepared as 1 M aqueous solutions. The aqueous solutions of (CH_3_COO)_2_Cu and CH_3_COOAg were prepared at concentrations of 0.25 M and 0.0625 M, respectively. Gaseous H_2_S was prepared from a dilute aqueous solution of NaHS.nH_2_S. Two milliliters of aqueous solution containing 10^−5^% NaHS.nH_2_S and the appropriate chemical compound was added to individual 15 mL tubes, which were sealed and incubated at room temperature for 5 min. Then, 1 mL of the gas phase was collected and measured by gas chromatography (model GC2014; Shimadzu Works, Kyoto, Japan). To determine which chemical compounds inhibited H_2_S volatilization more strongly, the minimum concentrations of H_2_S volatilization inhibition were determined using serial dilution methods. All test reagents were purchased from WAKO Pure Chemical Industries, Ltd. (Kyoto, Japan). The experiments were repeated at least three times.

### Inhibitory effects of zinc ions on the growth of oral bacteria

The bacterial strains used in the study are *P. gingivalis* FDC 381, *P. gingivalis* W83, *P. gingivalis* ATCC 33277, *F. nucleatum* ATCC 25586, *P. intermedia* ATCC 25611, *Streptococcus mutans* JCM 5705, *S. sobrinus* JCM 5176, *S. salivarius* GTC 0215, and *Streptococcus anginosus* FW73. The *S. mutans, S. sobrinus, S. salivarius,* and *S. anginosus* strains were cultivated in BD Bacto^TM^ brain heart infusion (BHI) medium (Becton, Dickinson and Company, Franklin Lakes, NJ, USA), while *P. gingivalis, F. nucleatum,* and *P. intermedia* were cultivated in BHI medium with hemin (5 μg/mL) and vitamin K (1 μg/mL).

Bacterial cultures were incubated at 37°C anaerobically until full growth, then suspended in fresh BHI medium or fresh BHI medium with hemin and vitamin K to an optical density at 600 nm (OD_600_) of 0.3. To evaluate the effect of zinc ions on the growth of bacteria, 100 μL of the inoculated medium was cultivated anaerobically in a final volume of 200 μL of ZnCl_2_-containing BHI medium or BHI medium with hemin and vitamin K. The final concentrations of ZnCl_2_ in the culture solutions were 0.1% (0.007 M), 0.01% (0.0007 M), 0.001% (0.00007 M), and 0% (control, 0 M). Cultivation was performed in a 96-well (flat-bottomed) microtiter plate (Nunc A/S, Roskilde, Denmark). After suspending the bacterial cells by pipetting, the optical density at 600 nm (OD_600_) was measured at 0 h, 12 h, and 24 h for Gram-positive bacteria and at 0 h, 12 h, 24 h, and 48 h for Gram-negative bacteria. The experiments were repeated at least three times.

### Statistical analysis

The Mann-Whitney *U*-test was used to evaluate the direct inhibitory effects of metal ions on gaseous H_2_S compared with 10^−5^% NaHS.nH_2_S solution, and the inhibitory effects of zinc ions on the growth of oral bacteria compared with that in the 0% ZnCl_2_ medium. Differences were considered to be significant when P<0.05. Statistical evaluations were carried out using the R software package, ver. 3.4.0 (http://www.R-project.org).

## Results

### Direct inhibitory effects of metal ions on hydrogen sulfide

**Figure 1:**
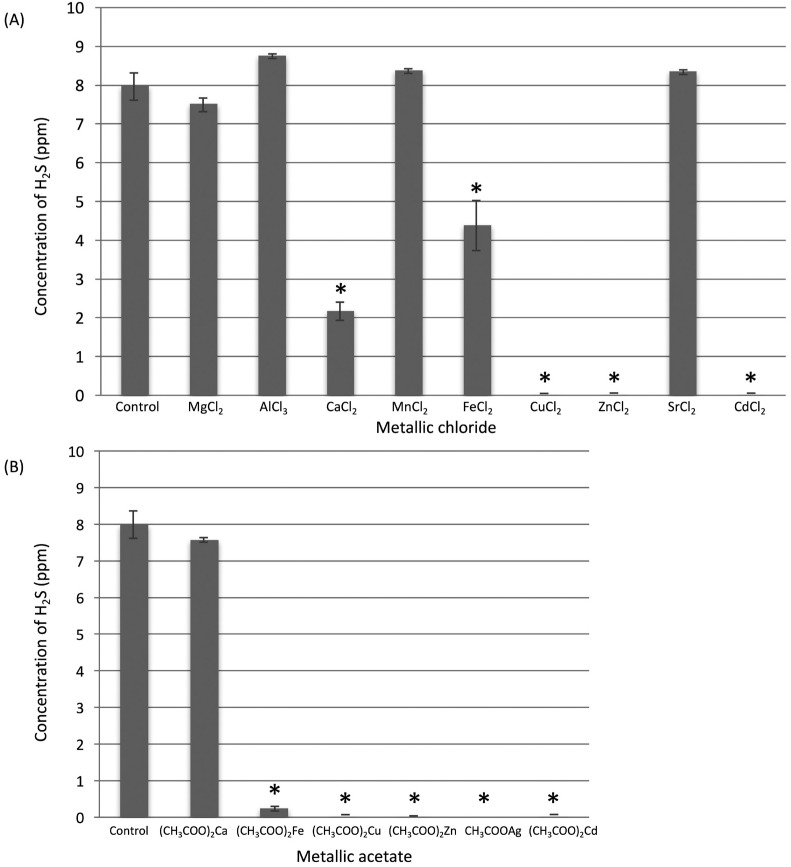
The direct inhibitory effects of aqueous solutions of metal chlorides (A) and metal acetates (B) on gaseous H_2_S

**Figure 2:**
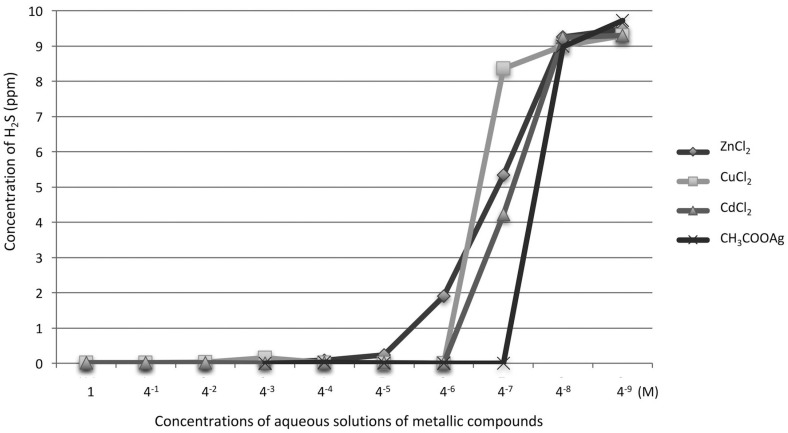
Determination of the minimum concentrations for inhibition of H_2_S volatilization using serially diluted aqueous solutions of ZnCl_2_, CuCl_2_, CdCl_2_, and CH_3_COOAg. The strength of H_2_S volatilization inhibition was in the following order: Ag+ > Cu_2_+ > Cd_2_+ > Zn_2_+

The inhibitory effects of metal chlorides on gaseous H_2_S are shown in Figure 1A. Aqueous solutions of ZnCl_2_, CuCl_2_, and CdCl_2_ almost entirely inhibited H_2_S volatilization. The CaCl_2_ and FeCl_2_ solutions moderately inhibited H_2_S volatilization, at 72.7% and 45.0%, respectively. There was a statistically significant difference between each of these five metal chlorides and the control (P<0.05). Aqueous MgCl_2_, AlCl_3_, MnCl_2_, and SrCl_2_ solutions did not inhibit H_2_S volatilization. For metal chlorides that directly inhibited gaseous H_2_S, as well as hydrophobic silver chloride, we assessed the inhibitory effects of the metal acetates of the same ions on gaseous H_2_S (Figure 1B). The (CH_3_COO)_2_Zn and CH_3_COOAg solutions inhibited H_2_S volatilization entirely (100% and 100%) (P<0.05), while (CH_3_COO)_2_Fe, (CH_3_COO)_2_Cu, and (CH_3_COO)_2_Cd inhibited it almost entirely (97.0%, 99.8%, and 99.9%) (P<0.05). Overall, four ions, zinc, copper, cadmium, and silver, were considered to have excellent inhibitory effects on H_2_S volatilization. Serially diluted aqueous solutions of ZnCl_2_, CuCl_2_, CdCl_2_, and CH_3_COOAg were assessed to determine the minimum concentration that inhibits H_2_S volatilization (Figure 2). The CH_3_COOAg solution exhibited the strongest effect, as it inhibited H_2_S volatilization entirely at 4^-7^ M. Comparing the strength of H_2_S volatilization inhibition among metals, the order was as follows: Ag^+^ > Cd^2+^ > Cu^2+^ > Zn^2+^.

* P<0.05 compared with the 10–5% NaHS.nH_2_S solution (Mann-Whitney U-test).

### Inhibitory effects of zinc ions on the growth of oral bacteria

**Figure 3:**
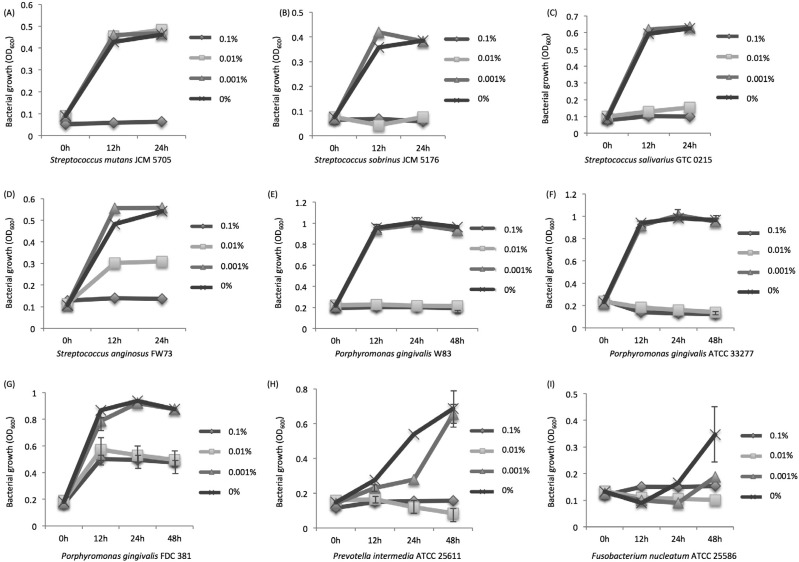
Inhibitory effects of zinc ions on the growth of oral bacteria (mean ± SD). *Streptococcus mutans* JCM5705 (A), *Streptococcus sobrinus* (B), *Streptococcus salivarius* GTC0215 (C), *Streptococcus anginosus* FW73 (D), *Porphyromonas gingivalis* W83 (E), ATCC 33277 (F), FDC 381 (G), *Prevotella intermedia* ATCC 25611 (H), and *Fusobacterium nucleatum* ATCC 25586 (I)

Among the tested oral bacteria, *F. nucleatum* ATCC 25586 was the most sensitive, as it was suppressed by 0.001% zinc ions in the medium (Figure 3). The growth of *S. sobrinus* JCM 5716, *S. salivarius* GTC 0215, *P. gingivalis* ATCC 33277, *P. gingivalis* W83, and *P. intermedia* ATCC 25611 was suppressed in medium with 0.01% zinc ions. For *P. intermedia* ATCC 25611, medium with 0.001% zinc ions suppressed bacterial growth for the first 24 h. The growth of *S. mutans* JCM 5705 was suppressed in medium with 0.1% zinc ions, but not in medium with 0.01% zinc ions. Although the growth of *S. anginosus* FW73 was suppressed in media with 0.1% and 0.01% zinc ions, the inhibitory effect of 0.01% zinc ions was not complete. *P. gingivalis* FDC 381 increased in the first 12 h in medium with 0.1% zinc ions as well as 0.01% zinc ions, but exhibited no further growth at 24 h and 48 h.

## Discussion

Among the nine metal ions examined in this study, calcium, iron, zinc, cadmium, copper, and silver had direct inhibitory effects on H_2_S volatilization. These metals have been widely used in dental materials, except for cadmium, which can have harmful effects on health. Iron, zinc, copper, and silver are not only used alone in tooth restorations but are also mixed into alloys due to their antimicrobial nature^1^ ^,^ ^8^ ^,^ ^10^ ^,^ ^23^. Calcium and silver are also used as antibacterial fillings for intractably infected root canals^4^. Iron ions have an inhibitory effect on glycosyltransferase enzymes of *S. mutans,* and a previous study reported that iron-containing sucrose reduced the amount of *mutans* streptococci in biofilm compared with sucrose^28^. For inhibition of odor in the oral cavity, zinc has been extensively studied and developed, whereas other metal ions have not been evaluated for this purpose. However, these metal ions exhibited direct inhibitory effects on H_2_S volatilization in this study, in addition to their previously reported antimicrobial effects. In particular, the inhibitory effects of copper and silver on H_2_S volatilization were excellent compared with that of zinc. The effects of these metal ions released from dental materials might thus contribute to reduce oral malodor due to antimicrobial effects and inhibition of H_2_S volatilization. Liu, et al.^10^ (2016) reported that Ti-Cu alloy implants released about 0.014 μg/day of Cu^2+^ ions, which is considerably lower than the minimal inhibitory concentration of Cu^2+^ for *Staphylococcus aureus* and *Escherichia coli.* The authors also explored the antibacterial activities of Ti-Cu alloy against *P. gingivalis* and *S. mutans,* and postulated that Ti-Cu alloys might exhibit antimicrobial activity markedly even if low levels of Cu^2+^ ions are released.

The ZnCl_2_ solution inhibited H_2_S volatilization entirely at 4^-4^ M (0.053%) in the current *in vitro* experiment. Young, et al.^28^ (2001) reported approximately 80% reduction of oral hydrogen sulfide after 1 h in a clinical trial of rinsing with 0.1% ZnCl_2_ solution. In other previous studies, zinc ions were usually combined with other antimicrobial agents, such as chlorhexidine, cetylpyridinium chloride, or triclosan^14^ ^,^ ^18^ ^,^ ^29^, and the concentrations of zinc compounds were in the range of 0.14% to 0.4%, which were effective for direct inhibition of H_2_S volatilization in an *in vitro* study. The recommended intake and tolerable intake of zinc established by the Food and Agriculture Organization of the United Nations and the World Health Organization (FAO/WHO) are 14-20 mg/day and 0.3-1 mg/kg body weight/day, respectively^26^, and therefore effective and safe concentrations of zinc ions can be incorporated into mouthwash. On the other hand, Japanese law requires the concentrations of some synthetic antimicrobial agents combined with dentifrice or mouthwash to be lower than their effective concentrations due to occurrences of anaphylactic shock^15^. For example, chlorhexidine and cetylpyridinium chloride are limited to 0.05% and 0.01%, respectively, although foreign studies examining the effectiveness of these compounds on oral malodor have been performed using 0.1%-0.2% chlorhexidine and 0.05%-0.07% cetylpyridinium chloride^2^ ^,^ ^25^. Interestingly, it has been reported that mouthwash containing 0.3% zinc acetate and 0.025% chlorhexidine showed a synergistic anti-VSC effect^30^. By combining multiple components, the concentration of each can be kept low. Furthermore, this synergistic effect indicates different mechanisms of VSC inhibition by each component.

The effect of zinc ions on the growth of oral bacteria was strain-dependent. *F. nucleatum, P. intermedia,* and *P. gingivalis* have been recognized as VSC-producing organisms that are important to oral malodor^21^. *F. nucleatum* and *P. intermedia* were especially sensitive to zinc ions compared with other bacteria assessed in this study. The growth of *F. nucleatum* ATCC 25586 and *P. intermedia* ATCC 25611 was suppressed entirely and for 24 h, respectively, with 0.001% ZnCl_2_. The growth of *P. gingivalis* W83 and ATCC 33277 was entirely suppressed by 0.01% ZnCl_2_, whereas strain FDC 381 increased for the first 12 h at both 0.01% and 0.1% ZnCl_2_ and then stopped growing. Gram-negative strict anaerobes have been identified as the main organisms capable of producing H_2_S, but *S. anginosus,* which is a Gram-positive microaerophilic anaerobe, has a greater capacity to produce H_2_S from L-cysteine than other oral streptococci^27^. The growth of *S. anginosus* FW73 increased for the first 12 h with 0.01% ZnCl_2_ and then stopped. The growth of *S. mutans* JCM 5705, *S. sobrinus* JCM 5176, and *S. salivarius* GTC 0215, considered unimportant for H_2_S production, was suppressed by 0.1% ZnCl_2_, 0.01% ZnCl_2_, and 0.01% ZnCl_2_, respectively. In a previous study on the effects of silver ions on the growth of *S. aureus* and *E. coli* ^9^, sensitivity may have been affected by the thickness of the peptidoglycan layer, which may inhibit ion passage through the Gram-positive bacterial cell wall. Differences in the zinc sensitivities of *P. gingivalis* strains should be investigated in the future.

This study determined that zinc ions inhibit oral malodor by two mechanisms, direct inhibition of H_2_S volatilization and antimicrobial activity. Both mechanisms may be caused by the strong affinity for the thiol groups characteristic of metal ions, including zinc ions. The direct reaction experiment between gaseous H_2_S and metal ions verified the first mechanism. Concerning the second mechanism, it has been reported that sulfur-containing amino acids such as cysteine and glutathione neutralized the activity of silver ions on bacteria^5^ ^,^ ^13^. This finding shows that metal ions react with sulfur-containing intracellular and extracellular amino acids, causing functional failure of proteins and resulting in damage to bacterial cells. In addition, functional failure of proteins may lead to the production of reactive oxygen species. In fact, the antimicrobial action of silver ions increased in the presence of oxygen^5^, which suggests that the generation of reactive oxygen species is related to the antimicrobial activity of metal ions.

There are several limitations to this study. First, the main VSC compounds related to odor in the oral cavity are H_2_S and CH_3_SH. Only the effect of zinc ions on H_2_S production was evaluated, and the effect on CH_3_SH should also be assessed. Second, no sustainability assessment of the inhibitory effect of zinc ions on H_2_S volatilization was performed in this study. A previous clinical trial reported that the inhibitory effect of rinsing with 0.1% ZnCl_2_ on H_2_S continued for only 1 hour, less than those of SnF_2_ and CuCl_2_ ^28^. The longitudinal evaluation of the binding reaction between zinc ions and H_2_S should be performed *in vitro.* Finally, the antimicrobial mechanism of zinc ions has been compared to the characteristics of silver ions, which have been studied well. The reason why the effect of zinc ions is weaker than those of silver and copper ions remains unclear. Further investigations into the chemical reactivity between zinc ions and thiol groups as well as the specific active sites of antimicrobial activity of zinc ions are necessary.

In conclusion, zinc ions, which exhibit an inhibitory effect on VSCs and are incorporated into products for oral malodor prevention, employ the two mechanisms of direct binding with gaseous H_2_S and antimicrobial activity. In particular, the growth of bacteria related to VSC production was inhibited at a lower concentration of ZnCl_2_ compared with bacteria not related to VSC production.
